# Why Do Our Fillings Fail?

**Published:** 1878-11

**Authors:** I. D. Pearce

**Affiliations:** Iowa City


### THE DENTAL REGISTER.

VOL. XXXII.] NOVEMBER, 1878. NO 11.

### "WHY DO OUR FILLINGS FAIL?"

BY DR. I. D. PEARCE, IOWA CITY.

*Read before the Iowa State Dental Society.*

When you selected me as one of the essayists for this meeting, I began to look around for something to write about, and for some time was at a loss for a subject; not that there was scarcity of topics, but what could I write about was the question. And finally as this subject that I have selected was the center around which gathers so much thought and earnest inquiry, I decided to prepare some thoughts upon the question, Why do our fillings fail? This is a theme that has commanded the earnest solicitation of riper minds than the one before you; more gigantic thoughts have grappled with this question than will be manifested in this paper.

Why did this fail? is a question that has engaged the most serious attention of men in all departments of trade and science. When men build structures that fail to subserve the purposes for which they were designed, they very naturally ask themselves this question: Why did it fail? So with the man of science. When he finds that he has failed in some great problem, he looks over the ground again to find wherein he has made the mistake. It may be the man who administers to the sick: he has properly diagnosed the case and given the right medicine, but it fails to have the desired effect; and how very naturally comes the question, fraught with intense anxiety, why did it fail? So, pertinent also, is the question to us. We have just completed a fine gold filling; it has required skill, patience and hard work; we admire it; we love to linger about it and show it to our special friends; but after awhile it returns to us in some secure corner of the old pocket book; it had not subserved the purpose for which it was intended. Then the question again, why has it failed? Some one says the anchorage was not good; another says there may have been a leak somewhere; others say it was the result of "oral electricity," or electro-chemical action; still other reasons might be offered, showing that we are not all harmonious as to the primary cause of these failures. Hence we are not satisfied to rest. And thus it is that this question is the uppermost one in the mind of our specialty to-day; we are thus forced to think and search. This is right; it is the moving power; it sends us onward and upward. In fact, without investigation there would be no progress in science.

Have you ever seen the field of research more thoroughly plowed and dug over than now? Why is it? Men everywhere and in everything find that in order to stand abreast with the advancement in their own avocation in life, can no longer stand around with their hands in their pockets, and take in, simply by absorption, the life of their calling, but they are awakening to the fact they must go out and dig for themselves.

Hence, we see men everywhere shouldering their picks and tools and entering the field. Here they come in contact with the weapons of other men, and they clash, thought with thought; mind grapples with nature; new things are discovered; these lead to other, and, perhaps, severer contests; greater thoughts with still greater. And thus it goes on; each discovery leading to further research; until the mind of but small compass, being expanded little by little, is enabled to grapple with the gigantic questions in nature never at rest; never satisfied; still reaching out into the unfathomable abyss beyond for more light. As evidence of this and as an aid in solving the question before us, let us glance, for a moment, at our past. And we need go no further back than within the vivid recollections of some who sit here to-day. There were men, who, before the advent of hard gold and mallets, inserted gold fillings—after some fashion—that failed not; fillings that saved teeth; fillings that stood as living monuments of praise; attesting to the skill of the hand and sweated brow that made them. Some of these old fillings come to see us once in a while, simply, we suppose, to show us that they are yet in a perfect state of preservation, although they have had some severe contests in the battles for life, and have occasionally been struck by lightning. Do you say these are but temporary? Shame on the man who would call them such, simply to bolster up a petted theory of doubtful foundation. But while some poor fellow was working away at one of these old fillings, with the sweat of an August day rolling from his brow, and with anxiety drawn up to the highest tension (while he was forcing the gold into the cavity with elbow grease) for fear his plugger would slip and plow up the soft tissue, Genius suggested to him to use less hand pressure and hit his instrument with something. He instantly acted upon the suggestion; thought it all over; and thus sprang into use the mallet. With this we thought we could do better work; save our patients broken necks, and live longer. But then we quarrelled about the kind of a mallet we should use. Dr. B thought that A's mallet was too light; C increased the weight of B's; and thus it run, until we have steel and lead mallets of from six to twelve ounces weight, so heavy as to frighten the patients upon whom they are used; and we think justly so. But we did not yet save all the teeth; fillings still failed. But genius stepped to the front again, and suggested to the mind still reaching out for something, better to anneal the gold, when the different particles will weld together, and the whole become a solid, compact mass; impervious to the fluids of the mouth. And from this sprang into existence all forms of hard gold. Then we cried "Eureka," "Eureka," we will now save teeth; with this cohesive gold, and the mallet, we certainly can make fillings that will not fail. But we find they do fail.

And when we remember the extreme to which we have run with these things, we ask is it much wonder that fillings, made out of 240 gold, and with twelve ounce mallets, fail? Is it not astonishing that the teeth into which they go retain any vitality? We repeat: is it not remarkable that any of them survive? But these thoughts bring us back to the original question: Why do our fillings fail? Why can not we, with the lessons we have learned from the past; the advice and counsels of the hoary heads and withered brows, whose strength has been spent in the front ranks, and with the advantage of all improved appliances for facilitating and perfecting all our work, make fillings stick? And, it is to this question, the question of all questions, that all eyes are now turned. We may not be able to fully answer this question, we rather think our pen is not adequate to the task. We may not be able to suggest anything very new, for many of the reasons of failure that we may refer to will, perhaps, seem to be old ones that have been discussed by other and abler pens than ours. But you know the scriptures teach us to keep the truths continually before us lest we forget them. "Bind them about thy neck that they may be with thee continually."

Very many of the causes of failure we are all agreed upon, but there are some alleged reasons upon which we split in twain. And, in this dicussion, we necessarily come in contact with these mooted questions. And very prominently amongst these stands out the one labelled "Oral Electricity."

This question has been very ably discussed in various articles by Prof. Chase and others. And, while they, in the main of their investigations, agree, yet, in their conclusions, radically disagree. And upon this disagreement hangs the most vital feature of this whole question, to-wit: the relative merits of gold and other materials for preserving teeth. Chase, Palmer and Flagg conclude from certain experiments that gold—considered electrically—is the poorest preserver of teeth. While Bridgeman and others conclude, from the same class of experiments, and upon the same hypothesis, that gold is the best preseiver of teeth. This question very naturally divides itself into two sections: Thermo and Voltaic electricity. The first of these every dentist is more or less acquainted with, and has observed its manifestations in teeth containing large metallic fillings in close proximity to a live pulp.

The pain experienced when the tooth is subjected, to sudden changes of temparature, is due to an electric current, from the inequality with which gold and tooth, conduct heat and cold.

But it is the second division, "voltaic electricity" upon which we separate. This is the question: it is the entering wedge; not that there is no such thing as "oral electricity," for this must be admitted; indeed, to deny it a man must close his eyes to scientific research; for it is a fact in Chemistry, that will not be gainsayed, that when two substances,—even two chips—are submerged in a liquid, and one is decomposed more rapidly than the other, that eletricity is set in motion. But this question is so universally accepted by scientists, that its discussion in the light of these investigations, is but a waste or time,

And we simply observe, as we leave this feature of the question, that there may be, and probably is, a current of electricity generated in a tooth filled with gold, or any other metal, when the reaction of the saliva is acid; but of so small an amount, when the tooth is full of vitality, that it would require a very delicate galvanometer to detect it.

When we connect a tooth, under such circumstances, with a galvanometer, by the ordinary insulated copper wire, and the needle is deflected, do you say that this current is from gold and enamel? Or do you not rather say that it is a compound current sent out, from the combined action, upon the gold, copper and enamel? Then there are other difficulties to contend with: such as the different electrical conditions of the atmosphere, and the different relation of metalic substances to the galvanometer; that taken altogether, render the accuracy of the results very uncertain; so much so, that we place but little reliance in any experiments of this kind, unless they are conducted in an isolated room, with an instrument so arranged as to make due allowance for the electrical changes in the atmosphere, and free from all metallic influence, except such as was used in the experiment. And then, we should not allow any other metal to come in contact with the liquid except such as was used as the filling. With the result of such an experiment we might be satisfied.

But the vital feature of the question, we have just reached: Does electricity break down organized tissue, in the sense in which it is alleged? We are aware of the fact, that a current of electricity from a voltaic battery, is supposed to break down, destroy, and wholly remove from the body, growths, such as tumors; and that it is frequently resorted to, by physicians for that purpose. So also, are we aware of the fact, that electricity is used to increase the vital energies, and even in some cases, restore lost vitality. Turn which way you may, you can but see in all nature, the destructive influence of this element: yet you can not close your eyes to its energizing and vitalizing influence in things around you, and in you; indeed, it is maintained by some, that our very life, is dependent upon this principle.

But the advocates of the theory of "oral electricity," allege that the pits or leaks, around gold fillings, are the result of voltaic electricity: that the gold being in a negative state, and the enamel positive; coming in contact, in the presence of the saliva, the latent electricity is put in motion; and the gold being a good conductor, while the enamel is a bad one, the electricity is thus interrupted in its motion, at the line of contact, and burns out. But our Chemistry, teaches us, that in the voltaic battery, chemical decomposition preceeds electrical action, and we ask, how is this? We learn, that to have electrical action you must first have decomposition; and this follows the action of the affinity, that one substance has for some element contained in some other substance. And the moment you expose a substance to the deorganizing influence of some other substance, and decomposition commences, then you have the electrical manifestation; and not, until this force, we call chemical affinity acts, do you observe any such manifestations.

But we are met at this point, with the theory that chemical action is entirely dependent upon the different electrical conditions of the substances brought in contact. This may be so: we are not fully prepared to deny the allegation.

We are aware that stronger pens than ours, support the theory: but we have failed to find experiments, so conclusive as to fully satisfy us that the theory needs no more support. Let us for a moment, put this theory to a practical test. First, let us restate the premises: Chemical action depends wholly, upon the different electrical conditions of the substances brought in contact. That in every tooth filled with a metal, we have a voltaic battery ready for action: the gold negative; the enamel positive: the gold a good conductor; enamel a bad one: hence, when in operation the latent electricity is put in motion, which is interrupted at the line of contact, of gold and enamel, and burns out. Now, we believet he grounds are fairly staked out: therefore, keep them in mind, while we look at this experiment. And this one will be sufficient, for the result here obtained, under the same influence, would be obtained in a thousand others. This tooth was perfectly filled, and filed down, and polished, until, under a strong magnifying glass, there were seen no pits, bruised enamel, or imperfections whatever. It was then suspended in Squibbs acetic acid, of the strength of one ounce acid in twenty-three ounces pure water, where it remained undisturbed for fourteen days. In order to support these conclusions—at least the last one,—it must, when removed from the liquid, show the enamel eaten out into small pits, or a continuous groove, around the gold fillings where the electricity had burned out: thus showing a decomposition here, and not elsewhere; or a greater decomposition here than elsewhere.

But, the tooth presents no such an appearance; and out of quite a number of like experiments, we have found no such condition. But we do see in this one, and in all others, very striking evidence that the entire surface of the tooth was acted upon alike. So that we see in these tests no support for the second proposition at least: neither do we see any such manifestation, in all our observations of failing fillings in the mouth.

With gold fillings, we generally find the first signs of failure, manifested at some one or two small points; while the rest of the line of contact may be perfect. And to say that these are the result of a difference in the polarity of the gold and enamel, is to affirm a proposition, that, to say the least, is not very tenable. We believe that the experiments, and general observations will support us in the conclusion that they are not evidence of opposite polarity, but that they are proof of imperfect work; either in not thoroughly removing diseased dentine or enamel at such points, or an imperfect adaptation of the gold to the tooth, a misgüided plugger, or a failure to perfectly finish at such points. When these imperfections occur around gold fillings, acid food, during eating, will be forced into them: decomposition of such deposited substances takes place, and the chemical action upon the tooth structure is increased. That the latent electricity put in motion, by this chemical decomposition of tooth substance may still *further*, accelerate this disorganization, we are not prepared to deny; but there is in a living tooth a force, that most certainly, to some extent, counteracts this external influence. But this phase of the question we will not discuss in this paper: Dr. Campbell, in the Missouri *Dental Journal*, has very ably argued this point. But we wish to call attention for a few moments, to another class of experiments reported by Dr. Bridgemen, in support of the first proposition: that chemical decomposition is *wholly* due to opposite polarity. He says: "a stout copper wire placed in sulphuric acid, so that one end is immersed in the acid while the other end stands out in the atmosphere, and allowed to so remain for a given time, and then removed, shows the portion of the wire at the surface of the acid, corroded about half way through, while that portion of the wire *above* the acid was coated with sulphate of copper; but in greater quantity at or near the surface of the acid, and gradually diminishing as you remove from the acid upward: while that portion of the wire is immersed, is simply corroded into pits and furrows, gradually decreasing in extent as you remove from the surface downwards. He jumps at the conclusion that this is all due to the atmosphere, changing the polarity of the wire, making one end positive and the other negative; from the following: A similar wire is *wholly* submerged in the acid, so that no portion of its surface is exposed to the atmosphere, remains for many months, without loss, or any chemical action upon it whatever; but the moment the least part of its surface becomes exposed to the air by evaporation of the acid, chemical decomposition commences. The rationale of these experiments is, that the acid has no affinity for the copper, until the wire is first polarized. This looks like very conclusive evidence in support of the Electro-Chemical theory. But let us look at the same experiments on another hypothesis. First, we observe that the copper wire *wholly* submerged into sulphuric acid of normal temparature, remains indefinitely, without apparant change. But the moment you raise the temparature of that acid to boiling heat, *rapid* decomposition ensues, and in a short time the copper is all dissolved. If this was the result of the wire being polarized, what produced the polarization? Now suspend a copper wire *over* sulphuric acid of normal temparature, in an open bottle, so that the lower end of the wire will come within three sixteenths of an inch of the surface of the acid, and you will soon observe the sulphate of copper distributing itself over the surface of the wire, but in greater quantities at the lower end, and gradually diminishing as the top is approached.

Now, it will be noticed that this is just what is seen in so much of Bridgemen's wire as is *above* the acid. Now, the question again: was this wire, that did not touch the acid, polarized? or does this class of experiments depend on some other principle? It is well known that when sulphuric acid is mixed with water, that the mixture becomes quite hot: so when the surface of this acid is exposed to the moisture of the atmosphere, it boils off into a fine spray; and becomes diffused with the atmosphere, and the further the spray is removed from the hot surface of the acid, the greater is the diffusion, and the lower the temparature. It is also known that heat accelerates decomposition, and that some substances remain undecomposed until the temparature is raised, when decomposition immediately commences.

This we have seen fully demonstrated with the copper wire submerged in sulphuric acid. Now, with these facts before us, let us return to the experiments. Can not we see, that as there is the greatest heat at the surface of the acid, there must be the greatest decomposition at this point? Sufficient to cut the wire as has been shown? And the further the wire is removed from the heated surface of the acid, that it comes in contact with a more diffused spray? with less heat? and consequently with a more diffused sulphate deposit?

In these experiments it was also shown that the increased moisture of the atmosphere, increased deposit; that when they were conducted in a dry atmosphere, the deposit was slow, but when made on a wet day, the deposite was quite rapid. But since we have already taken up more time on this feature of the question, than we had intended, we shall not have time to discuss another class of experiments showing the relative loss in weight of different materials used for filling teeth in the presence of different liquids, reported by Prof. Chase.

We simply observe that we secured a set of delicate scales and entered upon this series for our own satisfaction, and conducted several experiments. But so far, we have failed to get results that fully satisfy us that the Prof.'s conclusions are correct. Sometimes our specimens, which were reduced to exactly the same size and weight, showed, in contact with gold, a greater loss than when in contact with Fletcher's alloy, and other filling materials. But again, two sections of dentine, cut from the same tooth, and reduced to the same size and weight, were suspended in Squibb's acetic acid of the strength of one oz. of acid in twenty-three oz. of pure water, each in separate bottles, but one in contact with gold while there was nothing with the other. Both remained the same length of time, five days—when they showed the same loss,—six centigrams; hence conclude, that the results we have obtained, are not sufficiently uniform to prove anything. But *one* thing *was* clearly proven: that, it is very difficult to obtain exactly, the same weight, of the same thing, three times in succession. Another thing is prominently brought out, in all our experiments: that as the strength of the liquids, into which the specimens are thrown is increased, so is the loss in weight increased.

Now, since these experiments, to which, we have called your attention, as well as some others to which we have not alluded, seem to sustain us in the conclusion, that chemical action is *not wholly* due to polarization; but that the electrical manifestations of the oral *voltaic* batteries, may be due to the chemical decomposition; and to the strength of the disorganizing agent is increased so is the rapidity of the decomposition increased; therefore inasmuch as the electro-chemical theory fails to furnish us with an adequate, primary cause for the decay around our fillings, let us turn our inquiry in another direction. It is generally claimed by those who oppose the electro-chemical theory, that our failures are chiefly due to a want of thoroughness. It needs no argument to satisfy any honest, observing dentist that herein lies the secret of very many, and perhaps the *most* of our failures. But for some years past we have been largely experimenting in the mouth, with such things as 240 gold, deeply serrated pluggers, heavy mallets and other trash that has proven unfit as agents for preserving teeth.

And while we would not in the least discourage the practice of experimenting where actual harm might not follow, yet, while we may have intended thoroughness in every case, may we not reasonably expect *some* failures to come from so many experiments? cannot we find a ready solution of this question in this one thing of itself? just for one moment, look at your early experience with cohesive gold: how many times you have failed to work it into a cavity as you wanted to: and how often you have wanted to hire a boy to swear for you—just a little—when you have been trying to lay the foundation for a filling with it; work as you might the thing would turn upside down in spite of you; and finally when the plug was finished you was not satisfied with it: you had failed to perfectly adapt it to the walls of the cavity, and you knew it: you thought at the time, that it would be better to remove it at once, and put in an old-fashioned plug, until you had learned more about this "sticking stuff;" but you got your fee, and suffered the plug to go out, thinking that if it ever failed the patient would return: but unfortunately, the case dropped into the hands of another dentist, who marked it down against you, and finally it turns up as an argument against gold as a filling material. Do you say that this is simply a freak of the imagination, and has no foundation in fact? How many of these things have returned to your own hands, when you *knew* that the trouble was just what we have indicated. Need we say more in order to satisfy the intelligent body before us, that many of our failures are due to this cause alone? Again, did it ever occur to you that there might be found sufficient cause for failure, in cases that we had not correctly diagnosed? or if we had, they happened to belong to certain neighborhoods, when we were just at that particular time, needing a little advertisement? and we thought we could make something shine that would stay three or four years, when, if it came out, we would be so boosted up as to be able to counteract the damaging influence on some hypothesis;—perhaps, "oral electricity. Then again, the public seem to have the impression, that our avocation is a very money making business. They wholly overlook the fact that if nine-tenths of the dentists in the land were to die in the ranks, wholly dependent upon their savings from the profession, they would go to a paupers grave, leaving their families to the cold charities of the public. But from the incentive to make money easy, what a delusin—men rush to our ranks who have no adaptability to our calling:

no stock in trade other than the one of making money out of a credulous public. That this is a source from which we see so many failures will scarcely be denied. But again, our business is, in many places, reduced to a kind of barter and traffic system, so that it has become quite fashionable for people to go shopping amongst the dentists, and the man who wil offer most for the least is the lucky fellow. Hence prices tumble, until a man who has a family can scarcely get enough out of his profits to support it and live as a man ought. Therefore he works rapidly; makes haste so as to get through with just so much work, and in making haste he makes waste. He thinks because this thing is cheap he is justified in giving it a little less care than he would otherwise bestow upon it. So we see failure coming from this source, and a number of others that we have not time to dwell upon, such as the improper use of the mallet, the improper use of gold, the use of deeply serrated pluggers, contour fillings in grinding surfaces of molars and bicuspids, and the manipulation of amalgam in filling the cavity. But we fancy we hear it said that we will not materially disagree upon these being sources from which we get many failures; but why is it that gold in the hands of the best experts, sometimes fails to preserve teeth? We ask, are they not sometimes caught—unaware, perhaps—making haste? Do they never make mistakes? Do they never experiment with new forms of gold in the mouth? But you say if these failures generally come from a lack of thoroughness, why are you not always thorough? We say this is no argument against gold as a filling material; simply because a man is not always thorough is no evidence that he may not be so. What then is the remedy? First, adaptability of the man to the business; then his thorough preparation; not that he shall simply obtain a diploma, by purchase or otherwise, and display a fine case of instruments in a dazzeling office; but that he shall thoroughly understand his business. And that he shall be thorough in the treatment of his cases, and very thorough, in getting into a cavity, and very thorough in getting out, with, whatsoever he may come out; and thorough in the finish after he gets out, for just at this point he may fail, though all else be perfect. Let him o reception of new forms of gold, or new principles or instruments, test them first, out of the mouth; and never approach a living tooth with them until he has mastered their working qualities. We would bury all trade and traffic, that stimulates competition in prices, and encourage competition in excellency of work at reasonable prices. We would make all men work slow; we would have the word printed in bold letters, and hung about their necks during all office hours; for there is a halo of glory always hovering around the productions of the slow, careful workman. We would throw away all pluggers with deep serrations; they are not necessary in working soft gold, and are a curse with hard gold. We would make men mild and gentle with the mallet; teeth have handed in their checks, given up the ghost, under the blows of this instrument of torture. We would make men use more soft gold in approximal cavities of molars and bicuspids. We would make men more thorough in tracing out fissures, and desist from so much contour in the grinding surfaces of molars and bicuspids. Nature sometimes does a fine job here, but many cases are found where a thorough dentist could surpass his excellency in making a surface that would resist the ravages of those influences that linger about these multiplied cusps to find out the little caverns that may have been overlooked. Finally, we would combine the virtues of the past with the excellencies of the present. With soft gold and the modern method of preparing cavities, we can make better fillings than we did twenty-five years ago. But we can not do without hard gold, it has a place in our art that we can not ignore. We can not cure all these diseases in the teeth and make it permanent with either one or the other any more than we can cure all ailments of the body with quinine. Hence, the dentist must be discreet; he must have judgment; if he simply works by rule, he is a failure; if he lacks the physiological and pathological information he is a failure and has mistaken his calling. And now, after we have thoroughly prepared a man, we would put him upon trial for five years and if he failed to bring forth good fruit we would rout him out of our camp.

Brethren, in conclusion, let us go hence, pledged to more thoroughness in all our work, to be better dentists, to be careful, gentle and kind. Let us strive to see who can best work and best agree. Ever remembering that the eye of an intelligent public is upon us, watching for the men who are intelligent in their profession, thorough in their work, which they perform with gentle hands and kind, soothing words, during painful operations; cleanly in their persons and apartments; temperate and chaste in all their habits.

